# Effects of Herbal Adaptogen Feed-Additive on Growth Performance, Carcass Parameters, and Muscle Amino Acid Profile in Heat-Stressed Modern Broilers

**DOI:** 10.3389/fphys.2021.784952

**Published:** 2021-11-24

**Authors:** Elizabeth S. Greene, Clay Maynard, Casey M. Owens, Jean-François Meullenet, Sami Dridi

**Affiliations:** ^1^Center of Excellence for Poultry Science, University of Arkansas, Fayetteville, AR, United States; ^2^Arkansas Agricultural Experiment Station, University of Arkansas System Division of Agriculture, Fayetteville, AR, United States

**Keywords:** heat stress, broilers, adaptogen, growth performance, meat quality

## Abstract

Heat stress has strong adverse effects on poultry production and, thereby, threats its sustainability, which energized scientists to search for innovative and effective solutions. Here, we undertook this study to evaluate the effects of in-feed herbal adaptogen (stress response modifier) supplementation on growth performances, meat quality, and breast amino acid profile in chronic cyclic heat-stressed broilers. Day-old male Cobb 500 chicks (*n* = 720) were randomly assigned, in environmental chambers (*n* = 12, 24 pens), to three diet-treatments: a three-phase corn-soybean based diet fed as such (Control, C), or supplemented with the herbal adaptogen at 500 g/1000 kg control diet (NR-PHY-500) or at 1 kg/1000 kg control diet (NR-PHY-1000). From d29 to d42, birds from 9 chambers were exposed to cyclic heat stress (HS, 35°C from 9:30 am-5:30 pm), however, the rest of the chamber were maintained at thermoneutral conditions (24°C, TN), which creates 4 experimental groups: C-TN, C-HS, NR-PHY-500HS, and NR-PHY-1000HS (6 pens/group, 168 birds/group). HS altered growth performance via depression of feed intake and body weight. Adaptogen supplementation stimulated feed intake and averaged 65.95 and 83.25 g better body weight and 5 and 10 points better FCR at low and high dose, respectively, compared to heat-stressed birds. This increase in body weight was mirrored in enhanced weights of body parts (breast, tender, wings, and legs). Adaptogen supplementation modulated also breast amino acid profile, pH, color, and quality. Together, these data suggested that adaptogen supplementation could be a promising solution to alleviate heat stress, however further in-depth investigation for its mode of action and its underlying mechanisms are warranted.

## Introduction

Poultry industry supports the livelihoods and food security of billions of people worldwide. Both poultry meat and egg are globally highly regarded as the most efficient protein sources, with high organoleptic quality, relatively inexpensive, and without religious taboos (Barroeta, 2007; Cavani et al., 2009; Marangoni et al., 2015). However, poultry production sustainability is facing several challenges from a steep projected increase in global demand for high animal protein quality and the need to adapt to the pressure on natural resource availability and environmental constraints. In fact and according to the Food and Agriculture Organization (FAO) of the United Nations (Food and Agriculture Organization [FAO], 2018) and to the 2019 World Population Prospect (United Nations, 2019), it is predicted that the world human population will increase by 10% over the next decade, reaching approximately 9.7 billion in 2050. This, in turn, is estimated to drive a significant rise in the demand for food (∼73% in meat and 58% in dairy products), which require greater animal production including poultry that is projected to double by 2050.

Doubling poultry production within “planetary boundaries” to feed the growing global population will be challenging. It is clear that climate changes drive the earth system into a much warmer state (Alley et al., 2005; Chen et al., 2011). Indeed, unusual warm season with widespread and more intense heat waves have increased markedly over the past decades, and are likely to be larger in the future (Mora et al., 2013). Modern broiler chickens are highly thermo-sensitive and cannot cope well with high environmental temperatures because they are covered with feathers, they have high core body temperature (∼40°C) and high metabolic activity, and they lack sweat gland (Settar et al., 1999; Caulfield et al., 2014; Nawab et al., 2018). The strong adverse effect of heat stress-induced by high environmental temperature on broilers are well documented. Heat stress alters bird’s well-being by inducing stress (Star et al., 2008; Gu et al., 2012), depressing feed intake (Flees et al., 2017; Rajaei-Sharifabadi et al., 2017), inducing thirst (Belay et al., 1993), causing immunosuppression (Ghazi et al., 2012; Monson et al., 2018), reducing performance (Quinteiro-Filho et al., 2010; Lara and Rostagno, 2013), and in extreme case increasing mortality rate by spiraling hyperthermia (Furlan et al., 1998). For example, the European heat wave of summer 2003 resulted in the death of more than one million chickens in France (Fouillet et al., 2008). Another heat wave in 2015 killed more than 17 million birds in India (Bhadauria et al., 2016). In addition to welfare and performance issue, heat stress is a significant economic burden to the industry (St-Pierre et al., 2003), and it is a major thrust of intense research effort to identify effective strategies to ameliorate heat stress productivity loss.

Seminal works and various nutritional and/or managerial strategies were applied to mitigate heat stress, yet the poultry productivity losses are still high during hot seasons. Although they are known for more than 60 years in traditional medicine (for review see Panossian and Wikman, 2011), the use of in feed-adaptogens in livestock just begins to gain popularity (Selvam et al., 2018). Adaptogens, plant extracts, also known as stress response modifiers, are defined as metabolic regulators, which increase the ability of an organism to adapt to environmental stressors and to avoid damage from such stressors (Wagner et al., 1994; Panossian and Wikman, 2009, 2010). We undertook the present study to assess the effect of the adaptogen NR-PHY-30 (Natural Remedies, Bengaluru, India) on growth performance and carcass quality in broilers exposed to chronic cyclic heat stress.

## Materials and Methods

### Ethics Statement

All animal experiments were approved by the University of Arkansas Institutional Animal Care and Use Committee (IACUC # 21050) and were in accordance with the recommendations in NIH’s *Guide for the Care and Use of Laboratory Animals*.

### Animals, Experimental Design, and Sampling

A total of 672 one day-old Cobb 500 male broiler chicks were neck tagged, individually weighed, and randomly housed in twelve environmentally controlled chambers in the Poultry Environmental Research Laboratory at the University of Arkansas Research Farm. Each chamber was divided into 2 floor pens covered with fresh shavings and equipped with separate feeders and drinkers. Each pen contains 28 birds. The experiment was conducted in a complete randomized design with three diet treatments: a three-phase corn-soybean based diet (Table 1) fed as such (Control, C), or supplemented with the herbal adaptogen NR-PHY-30 (Natural Remedies, Karnataka, India) at 500 g/1,000 kg control diet (NR-PHY-500) or at 1 kg/1000 kg control diet (NR-PHY-1000) according to the manufacturer’s recommendation. The adaptogen was added to the diet in the crumble starter (d0–14) and in the pelleted grower (d15–28) and finisher (d29–42) as recommended by the manufacturer. The composition of the herbal adaptogen is proprietary to Natural Remedies (Karnataka, India), but is a polyherbal formulation of pre-standardized and tested herbs containing *Ocimum sanctum*, *Withania somnifera*, and *Emblica officinalis*. Lighting schedule was 24 h light for the first 3 days, reduced to 23 h light:1 h dark d 4–7, and reduced further to 18 h light:6 h dark thereafter. Ambient temperature was maintained as follows: 32°C for the first 3 days, then gradually reduced approximately by 3°C each week until it reached 24°C on d 21. On day 28, the temperature was increased daily in nine chambers to 35°C for 8 h per day (9:30 am to 5:30 pm) to create a cyclic heat stress pattern and mimic United States summer season until day 42. These chambers reached 35°C within 15 min of temperature adjustment. The three remaining chambers (six pens) were maintained at 24°C as a thermoneutral condition (TN). This creates 4 experimental groups (6 pens/group, 168 birds/group): birds fed C diet and maintained at TN (C-TN), birds fed C diet and exposed to heat stress (C-HS), birds fed NR-PHY-500 and exposed to heat stress (NR-PHY-500HS), and birds fed NR-PHY-1000 and exposed to heat stress (NR-PHY-1000HS). Before the onset of heat stress, two birds per pen were randomly selected and equipped with a Thermochron temperature logger (iButton, Embedded Data Systems, KY) for continuous monitoring of core body temperature as previously described (Rajaei-Sharifabadi et al., 2017). The environmental temperature and relative humidity were also continuously recorded in each chamber, inside and outside the barn. Feed intake and water consumption were recorded daily, and body weights were measured weekly. On day 41, after blood sampling, thermologger-equipped birds were humanely euthanized via cervical dislocation and tissues samples were collected, snap frozen in liquid nitrogen, and kept at –80°C for future use. On d42, the rest of birds were processed at the University of Arkansas Pilot Processing Plant (Fayetteville, AR, United States).

**TABLE 1:** Composition of basal diets (as fed basis, %)^1^.

	Period (Days)		
	
Ingredients (%)	Starter (0–14) Crumble	Grower (15–28) Pellet	Finisher (29–42) Pellet
Corn (7.81% CP)	60.53	60.99	66.52
Soybean meal (48% CP)	32.95	32.55	27.22
Poultry Fat (9000 kcal/kg)	1.80	2.38	2.46
Dicalcium Phosphate (18.5% P, 22% Ca)	2.08	1.85	1.67
Limestone (37% Calcium)	1.10	1.00	0.91
Sodium Chloride	0.38	0.40	0.44
DL-methionine (990 g/kg)^2^	0.38	0.30	0.27
L-lysine Hydrochloride (788 g L-lysine/kg)^3^	0.37	0.22	0.20
L-threonine (985g/kg)^4^	0.16	0.08	0.08
Choline Chloride (60%)	0.10	0.08	0.08
Vitamin/Trace Mineral Premix^5^	0.15	0.15	0.15
**Calculated Analysis (% unless specified)**			
ME (kCal/kg)	2994	3038	3108
Crude protein	21.71	21.30	19.18
Total phosphorus	0.77	0.71	0.66
Available phosphorus	0.45	0.42	0.38
Calcium	0.90	0.84	0.75
Chlorine	0.33	0.32	0.34
Sodium	0.16	0.17	0.19
Potassium	0.84	0.83	0.74
Methionine	0.67	0.59	0.54
Methionine + Cysteine	0.98	0.89	0.82
Lysine	1.32	1.18	1.04
Threonine	0.86	0.78	0.70
Linoleic acid	1.46	1.47	1.57
Dietary cation-anion balance	192	196	176

*^1^Treatments include: C-TN: birds raised under thermoneutral condition (24°C) from days 29 to 42 and had free access to the control diet; C-HS: birds exposed to cyclic high temperatures (8 h/d at 35^*o*^C; from 9:30 am to 5:30 pm) from days 29 to 42 and had free access to the control diet; NR-PHY-500HS: birds raised under the same condition as C-HS group, but fed the control diet supplemented with the herbal adaptogen at 500 g/1000 kg of diet, and NR-HPY-1000HS: birds raised under the same conditions as C-HS group, but fed the control diet supplemented with the herbal adaptogen at 1000 g/1000 kg of diet.*

*^2^Rhodimet^®^ NP9, ADISSEO, GA, United States.*

*^3^L-lysine HCl, AJINOMOTO HEARTLAND, INC. Eddyville, IA, United States.*

*^4^FENCHEM Ingredient Technology, Nanjing, China.*

*^5^Vitamins supplied per kg diet: retinol 3.33 mg, cholecalciferol 0.1 mg, α-tocopherol acetate 23.4 mg, vitamin K3 1.2 mg, vitamin B1 1.6 mg, vitamin B2 9.5 mg, niacin 40 mg, pantothenic acid 9.5 mg, vitamin B6 2 mg, folic acid 1 mg, vitamin B12 0.016 mg, biotin 0.05 mg, choline 556 mg. Minerals supplied per kg diet: Mn 144 mg, Fe 72 mg, Zn 144 mg, Cu 16.2 mg, I 2.1 mg, Se 0.22 mg.*

### Growth Performance

Birds were weighed individually on a weekly basis, while feed and water intake were measured on daily basis. Average body weight, average daily feed intake (individual and cumulative), and feed conversion ratio (FCR) were calculated for the experimental period (d 0–42).

### Mortality

Birds were monitored twice daily. For each dead bird, date, neck tag number, body weight and cause of death were recorded. This procedure continued throughout the study (up to d 42) to record mortality/treatment for each period thus allowing for adjusting performance parameters for daily mortality.

### Peripheral (Skin) Temperature

The surface temperature was measured by an Extech Flir I5 thermal imaging infrared camera (Extech Instruments, Long Branch, NJ, United States) in four birds per pen (24 birds/group) on d41 at 12 pm.

### Processing Parameters, Carcass Quality, and Meat Yield

Birds (∼150 birds/group) were processed at the University of Arkansas Pilot Processing Plant (Fayetteville, AR, United States) using a commercial inline system and carcass quality traits were determined as previously described (Orlowski et al., 2018). Briefly, birds were electrically stunned (11 V, 11 mA for 11 s), exsanguinated, scaled at 53.8°C for 2 min, and de-feathered using a commercial, inline equipment (Foodcraft Model 3; Baker international, MI, United States). Carcasses were manually eviscerated and rinsed before prechilling at 12°C for 15 min. Carcasses were, then, chilled for 90 min at 1°C in immersion chilling tanks with manual agitation at 15 min regular intervals. Slaughter weight, and prechilled carcasses were recorded, and following a 2 h chill at 4°C, the weight of breasts, tenders, leg quarters, wings, liver, and abdominal fat were recorded.

### Color

At time of debone, 24-h postmortem, and following cooking, intact left filets had color recorded with a handheld Minolta colorimeter and data was configured using SpectraMagic NX software (Minolta CM-400, Konica Minolta Sensing Americas Inc., Ramsey, NJ, United States), set with a 2-degree observer, decreasing surface reflectance, and illuminant parameters of D65. Before measuring, the colorimeter was calibrated to CIE specifications using a white calibration tile in agreeance with the procedure provided by the American Meat Science Association (AMSA) (American Meat Science Association [AMSA], 2012). Calibration values were entered according to the Y, x, and y calibration scheme (D65) and entered as 84.8, 0.3203, and 0.3378, respectively. Intact filets were positioned dorsal side up on white storage trays where measurements could be recorded on the left filet. Three separate L^∗^, a^∗^, and b^∗^ values were recorded for each filet in the cranial, medial, and caudal locations which were then subsequently averaged.

### pH

Immediately after color was recorded, pH of each left filet was measured. Left halves were also evaluated at 24-h postmortem for pH using a spear tip pH probe with automatic temperature compensation (Model 205, Testo instruments, West Chester, PA, United States). Samples were collected by inserting the pH probe near the wing joint area of each filet and allowed to equilibrate until a reading was maintained for 3 s.

### Water Holding Capacity/Drip Loss

Following part weight collection, filets were placed on white plastic storage trays, wrapped in plastic overlay liners, and placed in a walk-in cooler held at 4°C until 24-h postmortem. At 24-h postmortem, breast filets were removed from the cooler and reweighed for determination of drip loss. Drip loss percentage was calculated as a percent by weight in relation to deboned weight.

### Cook Loss

Deboned butterflies were trimmed of excess fat and any residual skin was removed. Butterflies were excised down the keel line and identification was kept on an individual basis for each left filet. Individual filets were then weighed to determine a precook weight. Eight filets of similar weight were cooked in aluminum foil covered pans (65 × 395 × 290 mm) on elevated baking racks in a commercial convection oven (Model E101-E, Duke Manufacturing Company, St. Louis, MO, United States) set to 176°C. A final end point temperature of 76°C (Model HT1000 thermometer, Cooper Instruments, Concord, ON, Canada) was reached before the filets were removed and allowed to cool at room temperature on white plastic storage trays. After cooling for approximately 1 h at room temperature, filets were reweighed to determine cook loss percentage. Cook loss was calculated as a percent, by weight, in relation to precook weight. Filets were then wrapped individually in aluminum foil sheets and were stored in refrigerated conditions (4°C) for approximately 24 h until instrumental texture analysis could be completed.

### Texture Analysis

Using a texture analyzer (Model TA-XT2 Plus, Texture Technologies, Scarsdale, NY, United States), tenderness was indirectly determined using the Meullenet–Owens Razor Shear (MORS), as described by Cavitt et al. (2004), during which MORS force (MORSF, N), MORS energy (MORSE, N.mm), and total peak counts (sums) were recorded. Briefly, a 5-kg load cell using a razor blade with a height of 24 mm and a width of 8.9 mm was set to a penetration depth of 20 mm. Crosshead speed was set at 5 mm/s and was triggered by a 5 g contact force. Data points were collected with an acquisition rate of 200 points per second. Breasts were punctured perpendicular to muscle fibers in four locations and shear energy was calculated as the area under the force deformation curve from the beginning to the end of the test.

### Measurement of Amino Acid Profile

Free amino acid assays of breast muscle tissues were carried out by Novus Analytical Service (St. Charles, MO, United States). Briefly, breast tissues were lyophilized for 48 h and milled to 1 mm particle size. Samples (200 mg) were accurately weighed into 50 mL digestion tubes and performic acid (2 mL) is added. The tubes are sealed and kept at 4°C for 16–24 h. Sodium metabisulfite (1.1 mL) is added to each sample, allowed to sit for 15 min followed by the addition of 11.9 mL 6N HCl. Samples were nitrogen purged, recapped, and placed in a 110°C oven for 24 h. The resulting hydrolyzates are neutralized along with internal standard addition. The samples are then filtered and analyzed on a Hitachi Amino Acid Analyzer using anion exchange chromatography followed by ninhydrin derivatization. For tryptophan measurement, samples (200 mg) were accurately weighed into 30 mL digestion tubes. 10 mL of nitrogen purged 4M NaOH was added to each tube along with 0.4 mL of 1M Dithiothreitol. Each tube is then purged with nitrogen, sealed, and placed in a 110°C oven for 22 h. Samples are neutralized with 0.22 M Sodium acetate then filtered for analysis. Tryptophan is separated using reverse phase chromatography and directly detected at 280 nm.

### Statistics

Data were analyzed by one-way ANOVA. In case ANOVA showed significant effects, the means were compared by Tukey’s multiple range test using Graph Pad Prism software (version 6.00 for Windows, Graph Pad Software, La Jolla, CA, United States). Data are expressed as the mean ± SEM, and means were considered statistically significant at a *P*-value ≤ 0.05.

## Results

**FIGURE 1:**
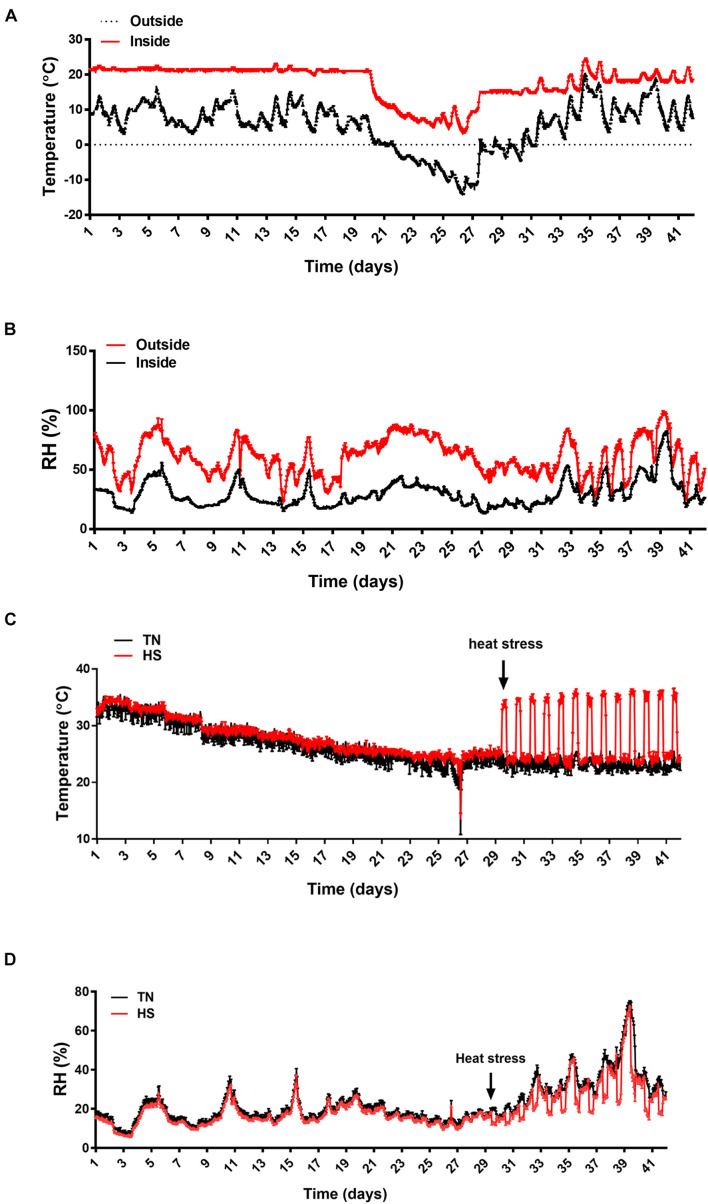
The environmental conditions during the experimental trial. **(A)** Temperature variations outside and inside the barn, **(B)** RH variations outside and inside the barn, **(C)** temperature variation inside the environmental chambers, and **(D)** RH variations inside the environmental chambers. Temperatures and RH were measured using thermologgers. RH, relative humidity.

The experiment has been conducted from January 21 to March 3, 2021 for 42 days. The weather cast (temperature and RH) is shown in Figures 1A,B. The average temperature was approximately 10°C and the RH was about 60–70%, which is typical in Arkansas during that season. The average temperature and the RH inside of the barn (but outside of the chambers) were ∼20°C and 30–40%, respectively (Figures 1A,B). The temperature inside of the environmental chambers was accurately manipulated to reach 35°C from 9:30 am to 5:30 pm and return to 24°C during the rest of the day each day from d29 to d42, creating a cyclic heat stress condition as planned (Figure 1C). The RH inside the chambers was ∼16–20% until d29 and then increased during heat stress to reach ∼45–60% (Figure 1D).

**FIGURE 2:**
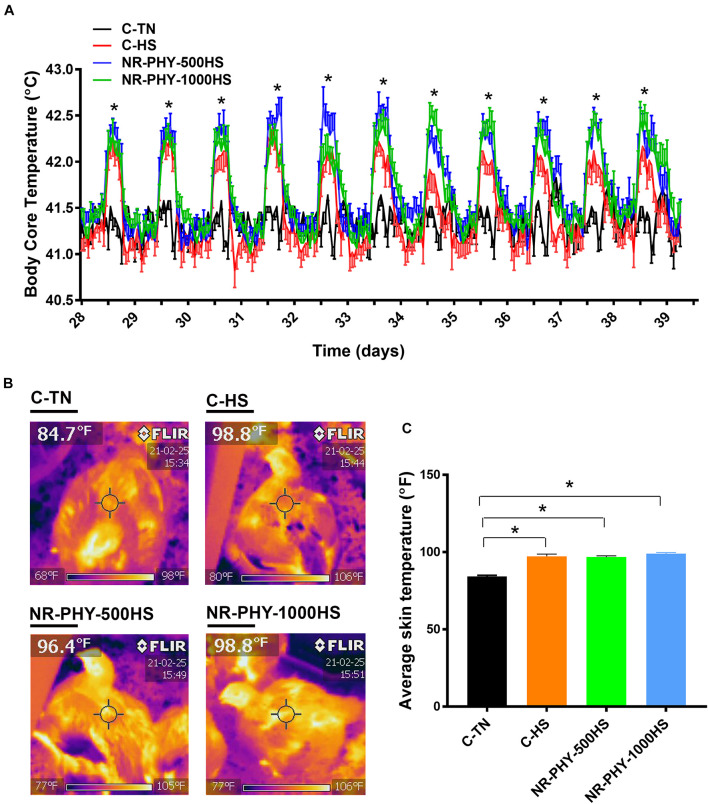
Effects of heat stress and adaptogen supplementation on surface and body core temperature. Core body temperature **(A)** was monitored using Thermochron temperature loggers (iButton, Embedded Data Systems, KY, United States) and surface temperature **(B,C)** was measured using an Extech Flir I5 thermal imaging infrared camera (Extech Instruments, Long Branch, NJ, United States). Data are mean ± SEM (*n* = 12/group for the body core temperature and *n* = 24 for the surface temperature). *Indicates a significant difference at P < 0.05. C, control diet; HS, heat stress; TN, thermoneutral.

As expected, heat stress significantly increased the broiler body core temperature by ∼0.5°C compared to TN conditions (Figure 2A). The supplementation of the herbal adaptogen (NR-PHY-30) increased further the body core temperature of heat-stressed broilers, although the difference was not statistically discernable (Figure 2A). Similarly, the infrared thermal imaging showed that the surface temperature was significantly higher in heat-stressed broilers fed with control- and adaptogen-supplemented diets compared to birds maintained at TN conditions (Figures 2B,C). There was no significant difference in the mortality rate between all groups (7.73, 6.54, 5.95, and 7.14% in C-TN, C-HS, NR-PHY-500HS, and NR-PHY-1000HS, respectively).

**FIGURE 3:**
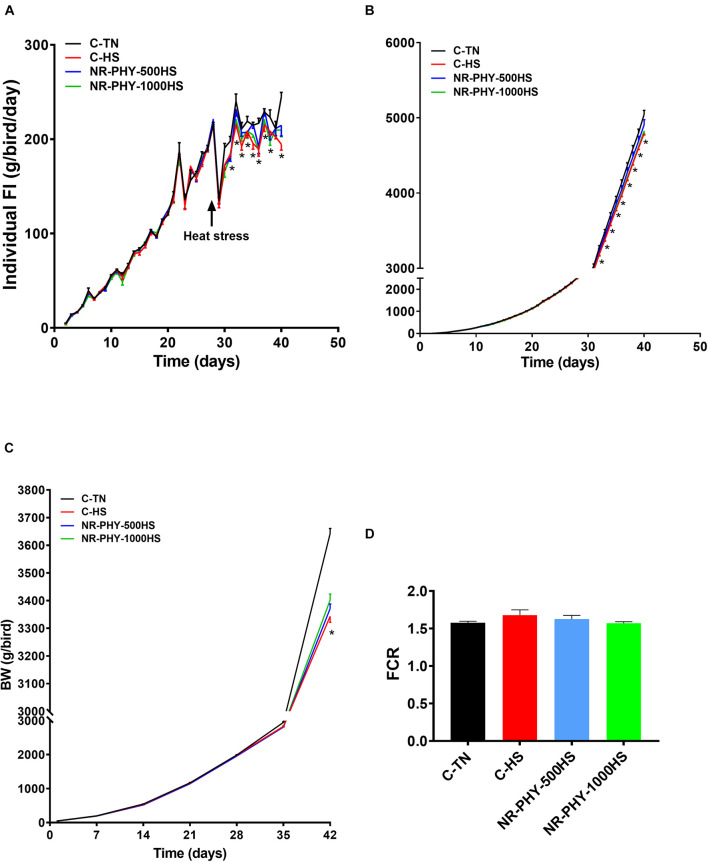
Effects of heat stress and adaptogen supplementation on growth performance. **(A)** Daily individual feed intake, **(B)** cumulative feed intake, **(C)** body weight, and **(D)** FCR. Pen and bird were experimental unit for feed intake and body weight, respectively. Data are mean ± SEM (*n* = 6/group for the feed intake, and *n* = 168 for body weight). *P < 0.05 compared to the control-TN.

As shown in Figures 3A–C, heat stress significantly reduced feed intake and body weight compared to TN conditions. Adaptogen supplementation significantly increased feed intake and body weight in a dose-dependent manner in heat-stressed broilers compared to those fed control diet and exposed to heat stress (Figures 3A,B). Adaptogen supplementation averaged 65.95 and 83.25 g better SW (Table 2) and 5 and 10 points better FCR at low (500 g/1000 kg diet) and high dose (1 kg/1000 kg diet), respectively, compared to heat-stressed birds (Figure 3D). Heat stress significantly reduced body part weights (HCW, CCWG, breast, tender, wings, and leg) (Table 2). Although it was not significant, except for tender, adaptogen supplementation increased, in a dose-dependent manner, breast weight (by ∼20 and 27.28 g), tender weight (by ∼2.66 and 5.18 g), and leg weight (by ∼7.42 and 15.94 g) at low and high doses, respectively, compared to heat-stressed birds fed control diet (Table 2).

**TABLE 2:** Effects of the herbal adaptogen on carcass and body parts weights and yields of heat-stressed broilers^1^.

	C-TN	C-HS	NR-PHY-500HS	NR-PHY-1000HS
SW (g)	3524.3720.82^a^	3186.1025.32^b^	3252.0218.91^b^	3269.3525.23^b^
HCW (g)	2610.2817.21^a^	2365.9820.41^b^	2408.7015.03^b^	2426.3920.50^b^
CCWG (g)	2674.9317.37^a^	2420.8220.67^b^	2465.3215.08^b^	2485.3520.98^b^
Fat (g)	38.670.93	36.130.88	35.180.82	37.001.05
Fat (%)	1.490.04	1.520.04	1.460.03	1.520.04
Breast (g)	764.367.36^a^	665.948.05^b^	685.926.84^b^	693.228.14^b^
Breast yield (%)	29.230.17^a^	28.070.19^b^	28.430.16^b^	28.520.18^b^
Tender (g)	139.991.29^a^	124.721.20^b^	127.381.08^bc^	129.901.42^c^
Tender yield (%)	5.350.03	5.270.03	5.290.03	5.350.03
Wings (g)	270.001.78^a^	249.072.15^b^	251.631.68^b^	249.252.11^b^
Wing yield (%)	10.350.05^a^	10.550.05^b^	10.460.04^ab^	10.290.05^a^
Leg (g)	766.505.67^a^	715.237.13^b^	722.654.73^b^	731.176.99^b^
Leg yield (%)	29.360.13^a^	30.230.15^b^	30.030.14^b^	30.140.14^b^

*^1^Data are means ± SEM (*n* = 150 birds/group). Treatments are: C-TN: birds raised under thermoneutral condition (24°C) from days 29 to 42 and had free access to the control diet; C-HS: birds exposed to cyclic high temperatures (8 h/d at 35^*o*^C; from 9:30 am to 5:30 pm) from days 29 to 42 and had free access to the control diet; NR-PHY-500HS: birds raised under the same condition as C-HS group, but fed the control diet supplemented with the herbal adaptogen at 500 g/1000 kg of diet, and NR-HPY-1000HS: birds raised under the same conditions as C-HS group, but fed the control diet supplemented with the herbal adaptogen at 1000 g/1000 kg of diet CCWG, chilled carcass without giblet; HCW, hot carcass without giblet; SW, slaughter weight. Different letters within a row indicate a significant difference at *P* < 0.05.*

Heat stress did not affect the pH of breast meat at both processing and 24 h postmortem (Table 3). However, adaptogen supplementation at both doses significantly increased pH at processing only, and not at 24 h postmortem (Table 3). Heat stress significantly reduced breast meat b^∗^ value at processing, and did not affect any other color value (Table 4). Breasts from adaptogen-fed birds exhibited significant higher processing L^∗^ and b^∗^ values compared to those from heat-stressed birds fed a control diet (Table 4). Meat color parameters (L^∗^, a^∗^, and b^∗^) did not differ between all groups at 24 h postmortem and after cook (Table 4). Cook loss was significantly reduced by heat stress, and increased to normal levels by adaptogen supplementation (Table 5). Adaptogen supplementation at high dose (1 kg/1000 kg diet) significantly increased MORSE compared to birds fed control diet and maintained under both TN and heat stress conditions (Table 5).

**TABLE 3:** Effects of the herbal adaptogen on breast meat pH of heat-stressed broilers^1^.

	C-TN	C-HS	NR-PHY-500HS	NR-PHY-1000HS
At processing	6.100.01^a^	6.130.01^a^	6.190.01^b^	6.190.01^b^
24 h postmortem	5.980.02	6.030.02	5.990.02	6.050.02

*^1^Data are means ± SEM (*n* = 150 birds/group). Treatments are: C-TN: birds raised under thermoneutral condition (24°C) from days 29 to 42 and had free access to the control diet; C-HS: birds exposed to cyclic high temperatures (8 h/d at 35^*o*^C; from 9:30 am to 5:30 pm) from days 29 to 42 and had free access to the control diet; NR-PHY-500HS: birds raised under the same condition as C-HS group, but fed the control diet supplemented with the herbal adaptogen at 500 g/1000 kg of diet, and NR-HPY-1000HS: birds raised under the same conditions as C-HS group, but fed the control diet supplemented with the herbal adaptogen at 1000 g/1000 kg of diet. Different letters within a row indicate a significant difference at *P* < 0.05.*

**TABLE 4:** Effects of the herbal adaptogen on breast meat color of heat-stressed broilers^1^.

	C-TN	C-HS	NR-PHY-500HS	NR-PHY-1000HS
**At processing** L*	52.75 ± 0.18^ac^	52.32 ± 0.18^a^	53.68 ± 0.16^b^	53.12 ± 0.20^bc^
a*	3.23 ± 0.10	2.93 ± 0.09	3.13 ± 0.08	3.14 ± 0.10
b*	8.62 ± 0.10^a^	8.18 ± 0.11^b^	8.76 ± 0.10^a^	8.66 ± 0.10^a^
**24 h Postmortem** L*	54.87 ± 0.49	54.04 ± 0.49	55.09 ± 0.34	55.59 ± 0.36
a*	2.72 ± 0.17	3.04 ± 0.22	2.96 ± 0.17	3.22 ± 0.21
b*	8.25 ± 0.19	8.18 ± 0.24	8.47 ± 0.017	8.83 ± 0.30
**Post cook** L*	77.51 ± 0.67	77.87 ± 0.53	76.87 ± 0.75	76.61 ± 0.39
a*	3.58 ± 0.22	4.00 ± 0.21	4.13 ± 0.26	3.84 ± 0.12
b*	20.32 ± 0.44	19.98 ± 0.62	21.34 ± 0.64	20.86 ± 0.47

^1^Data are means ± SEM (*n* = 150 birds/group). Treatments are: C-TN: birds raised under thermoneutral condition (24°C) from days 29 to 42 and had free access to the control diet; C-HS: birds exposed to cyclic high temperatures (8 h/d at 35°C; from 9:30 am to 5:30 pm) from days 29 to 42 and had free access to the control diet; NR-PHY-500HS: birds raised under the same condition as C-HS group, but fed the control diet supplemented with the herbal adaptogen at 500 g/1000 kg of diet, and NR-HPY-1000HS: birds raised under the same conditions as C-HS group, but fed the control diet supplemented with the herbal adaptogen at 1000 g/1000 kg of diet. Different letters within a row indicate a significant difference at *P* < 0.05. L*, lightness; a*, redness and b*, yellowness.

**TABLE 5:** Effects of the herbal adaptogen on breast meat drip and cook loss^1^.

	C-TN	C-HS	NR-PHY-500HS	NR-PHY-1000HS
Drip Loss (g)	4.131.52	4.750.71	5.610.89	7.541.17
Cook Loss (g)	79.213.63^a^	57.542.74^b^	79.135.95^a^	86.133.94^a^
MORSF (N)	12.400.39	12.510.39	13.390.56	13.940.43
MORSE (N*mm)	165.345.99^a^	166.274.97^ab^	179.087.13^ab^	187.896.32^b^
Peak Counts	8.020.32	8.390.40	8.150.27	7.720.32

*^1^Data are means ± SEM (*n* = 150 birds/group). Treatments are: C-TN: birds raised under thermoneutral condition (24°C) from days 29 to 42 and had free access to the control diet; C-HS: birds exposed to cyclic high temperatures (8 h/d at 35°C; from 9:30 am to 5:30 pm) from days 29 to 42 and had free access to the control diet; NR-PHY-500HS: birds raised under the same condition as C-HS group, but fed the control diet supplemented with the herbal adaptogen at 500 g/1000 kg of diet, and NR-HPY-1000HS: birds raised under the same conditions as C-HS group, but fed the control diet supplemented with the herbal adaptogen at 1000 g/1000 kg of diet. Different letters within a row indicate a significant difference at *P* < 0.05. MORSF, Meullenet-Owens razor shear force, and MORSE, Meullenet-Owens razor shear energy.*

Heat stress did not elicit any change to free amino acid profile in breast tissues compared to TN environment in our experimental conditions (Table 6). Supplementation of adaptogen at both doses significantly reduced breast cysteine levels compared to heat-stressed birds fed with control diet (Table 6). High dose (1 kg/1000 kg diet) of adaptogen significantly decreased the breast levels of glutamic acid, glycine, serine, and threonine compared to heat-stressed birds fed with control diet (Table 6).

**TABLE 6:** Effects of the herbal adaptogen on breast muscle amino acid profile^1^.

Amino acids (%)	C-TN	C-HS	NR-PHY-500HS	NR-PHY-1000HS
Alanine	4.5360.07^a^	4.5360.07^a^	4.4080.05^ab^	4.2360.09^b^
Arginine	4.9260.07^a^	4.9180.08^ab^	4.770.06^ab^	4.6180.09^b^
Aspartic acid	7.4240.11	7.4280.11	7.1760.10	6.9480.15
Cysteine	0.9760.01^a^	0.9660.007^a^	0.9280.01^b^	0.9040.01^b^
Glutamic acid	11.9420.12^a^	11.9380.12^a^	11.50.18^ab^	11.0860.20^b^
Glycine	3.2080.01^a^	3.1840.03^a^	3.1520.02^a^	3.0060.05^b^
Histidine	2.6140.10	2.7040.14	2.6520.06	2.5980.09
Isoleucine	3.7240.06	3.7460.06	3.620.05	3.5080.08
Leucine	6.2260.09	6.2280.09	6.0180.09	5.8320.12
Lysine	8.1140.16	8.2040.18	7.8260.16	7.6320.19
Methionine	2.3040.04	2.330.04	2.250.03	2.1740.05
Methionine + Cysteine	3.2780.05	3.2940.05	3.1780.05	3.0780.07
Phenylalanine	4.9260.14	5.0160.20	4.8280.10	4.750.18
Proline	2.0620.02	2.0880.04	2.0340.04	2.010.07
Serine	3.1240.03^a^	3.1120.03^a^	2.9940.04^ab^	2.8820.05^b^
Threonine	3.5180.05^ab^	3.5340.05^a^	3.4060.05^ab^	3.30.07^b^
Tryptophan	0.8020.02	0.8140.01	0.8140.008	0.7620.02
Tyrosine	2.0420.04	2.0420.03	1.9820.04	1.9380.04
Valine	2.5520.04	2.4360.09	2.4840.03	2.4220.05

*^1^Data are means ± SEM (*n* = 6 birds/group). Treatments are: C-TN: birds raised under thermoneutral condition (24°C) from days 29 to 42 and had free access to the control diet; C-HS: birds exposed to cyclic high temperatures (8 h/d at 35°C; from 9:30 am to 5:30 pm) from days 29 to 42 and had free access to the control diet; NR-PHY-500HS: birds raised under the same condition as C-HS group, but fed the control diet supplemented with the herbal adaptogen at 500 g/1000 kg of diet, and NR-HPY-1000HS: birds raised under the same conditions as C-HS group, but fed the control diet supplemented with the herbal adaptogen at 1000 g/1000 kg of diet. Different letters within a row indicate a significant difference at *P* < 0.05.*

## Discussion

There is a monumental pressure on farm animals to increase production to feed the future and meet the growing global demand for high-quality animal proteins. Due to various constraints, including extreme environmental conditions, this will be very challenging. Heat load is one of the most challenging stressor to poultry industry worldwide because of its strong adverse effects on welfare, feed intake, growth, immunity, meat yield, and mortality (Star et al., 2008; Quinteiro-Filho et al., 2010; Ghazi et al., 2012; Lara and Rostagno, 2013; Flees et al., 2017; Rajaei-Sharifabadi et al., 2017; Monson et al., 2018; Liu et al., 2019). Over the past decades, widespread extreme heat waves have occurred repeatedly and caused great losses across the globe (Rotter and Van de Geijn, 1999; St-Pierre et al., 2003; Thornton et al., 2009; Nardone et al., 2010; Reynolds et al., 2010). These heat anomalies are projected to increase in the future and their strong adverse effects will likely take a heavy toll and large amplitude (Meehl and Tebaldi, 2004; Sherwood and Huber, 2010; Liu et al., 2017; Mora et al., 2017). There is, therefore, a critical need to identify new effective strategies (management, nutrition, etc.) to mitigate these adverse effects and ameliorate heat stress productivity losses.

Fueled by consumer preferences for natural products, there is growing interest in using phytobiotic feed additives in the animal food system (American Meat Science Association [AMSA], 2012). Although they are known for more than 60 years in medicine and pharmacosanation (Wagner et al., 1994), and their original plant sources have been used for centuries in traditional medicine (Wagner et al., 1994; Panossian and Wikman, 2011), adaptogens are just recently begin to be used as feed additive in poultry (Selvam et al., 2018; Marimuthu et al., 2020). Herbal adaptogens are plant-derived biologically active substances originally defined as stress-response modifiers and metabolic regulators that increase the ability of an organism to adapt to environmental stressors and, thereby, protect against cellular damage from such stressors (Duraisami et al., 2010; Giri et al., 2011, 2013; Singh et al., 2017). In this study, we sought to assess the effect of the herbal adaptogen NR-PHY-30, formulated by Natural Remedies Private Limited, Bengaluru, India, on growth performance, carcass quality and breast amino acid profile in heat-stressed broilers.

NR-PHY-30 contains Indian gooseberry (*Emblica officinalis*), holy basil (*Ocimum sanctum*), and winter cherry (*Withania somnifera*) (Natural Remedies, personal communication), and the adaptogenic properties of these plants have been already reported (Wagner et al., 1994; Panossian and Wikman, 2011) as well as their beneficial therapeutic effects (Pattanayak et al., 2010; Malik et al., 2016; Dutta et al., 2019). As expected and in agreement with previous studies, including our own (Flees et al., 2017; Rajaei-Sharifabadi et al., 2017; Liu et al., 2020; Tabler et al., 2020; Wasti et al., 2020), heat stress increased both surface and body core temperatures and reduced feed intake and body weight in modern broilers. Interestingly, although it increased further the surface and body core temperatures, the herbal adaptogen NR-PHY-30 enhanced feed intake and body weight and improved FCR in heat-stressed broilers. This suggests one of the two following potential scenarios: (1) While it is known that the body experiences a temperature-dependent variation in energy needs that should be reflected in feed intake (reduction under heat stress conditions) (Brobeck, 1948; Jakubczak, 1976), the effect of the adaptogen NR-PHY-30 on appetite and feed intake in our experimental conditions was probably independent from the thermostatic mechanism (Brobeck, 1948). For instance, it has been shown that intracerebroventricular administration of IL-1 raised body core temperature without affecting feed intake in rodents indicating that feed intake regulation could be uncoupled from the thermoregulatory mechanisms (McCarthy et al., 1986). (2) Although it was not measured in the present study, as the adaptogen was supplemented from d1 post hatch, it is probable that NR-PHY-30 increased the body core temperature at early age, which mimics a repeated mild exposure and low stress dose that resulted in increased resistance and better adaptation to heat stress (thermo-tolerance) at later age. This second scenario might be supported also by previous thermal conditioning studies showing that temporarily elevated brooding temperatures at early post-hatch or at embryonic age impart long-term resistance to heat stress in broilers, which survive and grow better at higher environmental temperatures (De Basilio et al., 2003; Gunal, 2013; Loyau et al., 2013, 2015; Zaboli et al., 2017).

The increased body weight was accompanied by an enhanced weights of body parts, including breast and tender weights along with a significant reduction in breast levels of free alanine, arginine, cysteine, glutamic acid, glycine, and serine. This indicates that the adapotogen NR-PHY-30 might stimulate amino acid incorporation and use by the muscle for protein synthesis. Previous studies have shown the key role of alanine (Perez-Sala et al., 1987), arginine (Wang et al., 2018), cysteine (Chua et al., 1984), glutamic acid (Brodsky et al., 2017), glycine (Wang et al., 2014), and serine (Galbraith and Buse, 1981) in protein synthesis. What downstream pathways used by the adaptogen to enhance muscle protein synthesis under heat stress condition is not known and merit further in depth investigation.

Although heat stress did not affect the breast meat pH, it reduced the b^∗^ (yellowness) value, which corroborated previous studies (McKee and Sams, 1997; Aksit et al., 2006). This might be associated with heat stress-induced disruption of muscle membrane integrity, denaturation of muscle proteins, and in turn increased light scattering (Owens et al., 2000; Sandercock et al., 2001). Interestingly, the adaptogen supplementation increased the pH at processing and increased the L^∗^ value, suggesting potential improvement of meat color. Although the underlying mechanisms are not known at this time, it is possible that the adaptogen supplementation improved the antioxidant status of the breast muscle (Arif et al., 2016), which in turn improve meat quality. Intriguingly, heat stress reduced cook loss and the adaptogen supplementation reverse this parameter to the same levels of breast from TN birds fed with control diet. Similarly, MORS total energy (MORSE) was not altered by heat stress, but increased with the adaptogen supplementation, indicating that the adaptogen reduced breast meat tenderness. Although it was not measured here, and in addition to collagen and tissue connective content in the breast, tenderness might be affected by the sarcomere length, muscle fiber diameter, stromal proteins, and collagen solubility (Koohmaraie et al., 2002), all of which might be modified by the adaptogen administration, which warrant further investigations.

## Conclusion

Supplementation of the herbal adaptogen NR-PHY-30 stimulated appetite and feed intake, and in turn improved growth performance in a dose-dependent manner (average 66–83 g in body weight and 5–10 points better FCR) in cyclic heat-stressed broilers, which make it a promising nutritional strategy. However, further in depth investigation are needed to define its mode of action and its underlying molecular mechanisms.

## Data Availability Statement

The original contributions presented in the study are included in the article/supplementary material, further inquiries can be directed to the corresponding author/s.

## Ethics Statement

The animal study was reviewed and approved by the University of Arkansas.

## Author Contributions

SD designed the experiment and purchased the reagents. EG and SD conducted the trial and processed the animals at the end of trial. EG and CM measured and analyzed the meat quality parameters. SD wrote the manuscript with input from EG, CM, CO, and J-FM. All the authors have read and agreed to the published version of the manuscript.

## Conflict of Interest

The authors declare that the research was conducted in the absence of any commercial or financial relationships that could be construed as a potential conflict of interest.

## Publisher’s Note

All claims expressed in this article are solely those of the authors and do not necessarily represent those of their affiliated organizations, or those of the publisher, the editors and the reviewers. Any product that may be evaluated in this article, or claim that may be made by its manufacturer, is not guaranteed or endorsed by the publisher.
